# Genome-Wide Identification and Analysis of MAPK and MAPKK Gene Families in Bread Wheat (*Triticum aestivum* L.)

**DOI:** 10.3390/genes8100284

**Published:** 2017-10-20

**Authors:** Haoshuang Zhan, Hong Yue, Xian Zhao, Meng Wang, Weining Song, Xiaojun Nie

**Affiliations:** 1 State Key Laboratory of Crop Stress Biology in Arid Areas, College of Agronomy and Yangling Branch of China Wheat Improvement Center, Northwest A&F University, Yangling 712100, China; zhanhaoshuang@nwsuaf.edu.cn (H.Z.); yuehongsx@163.com (H.Y.); jooxian568394@163.com (X.Z.); ours2010@163.com (M.W.); sweining2002@yahoo.com (W.S.); 2 Australia-China Joint Research Centre for Abiotic and Biotic Stress Management in Agriculture, Horticulture and Forestry, Yangling 712100, China

**Keywords:** abiotic stress, co-expression network, expression profiles, *Triticum aestivum*

## Abstract

The mitogen-activated protein kinase (MAPK) cascade is a universal signal transduction module that plays a vital role in regulating growth and development, as well as environmental stress responses in plants. Wheat is one of the most important crops worldwide. Although the MAPK kinase kinase (MAP3K) family in wheat has been investigated, the MAPK and MAPK kinase (MAP2K) gene families remain unknown at present. Here, 54 MAPK and 18 MAPKK genes were identified in wheat using recent genomic information. Phylogenetic analysis of *Triticum aestivum* L. MAPKs and MAPKKs (*TaMAPKs* and *TaMAPKKs*) together with homologous genes from other species classified them into four groups, and the clustering was consistent with the genomic exon/intron structures. Conserved motif analysis found that MAPK proteins contained a typical TXY phosphorylation site and MAPKK proteins contained an S/T-X5-S/T motif. RNA-seq data mapping analysis showed that MAPK and MAPKK genes in group IV had tissue-specific expression profiles, whereas each group I member showed relatively high expression in all organs. Expression patterns of *TaMAPK* and *TaMAPKK* genes under stress conditions were also investigated and stress-responsive candidates were identified. Co-expression network analysis identified 11 *TaMAPK* genes and 6 *TaMAPKK* genes involved in the interaction network pathway. Overall, this study provided useful information for evolutionary and functional surveys of MAPK and MAPKK gene families in wheat and beyond.

## 1. Introduction

Wheat is one of the most important cereal crops worldwide, and provides about 30% of the staple food source for humankind [1,2]. Various abiotic stresses, including salt, drought and cold, are major limiting factors to wheat production. Improving the tolerance of wheat in response to diverse stresses holds promise for addressing the ever-increasing human population as well as the threat of climate change.

The self-protection mechanisms of plants activate numerous genes during enduring abiotic stress [3]. Mitogen-activated protein kinase (MAPK) cascade genes code for well-conserved proteins that function as key signal transduction components, consisting of three MAPK members activated by sequential phosphorylation, namely (MAPK kinase kinase) MAP3K- (MAPK kinase) MAP2K-MAPK [4]. Extensive studies have revealed that the MAPKs have indispensable regulatory roles in response to abiotic stresses in plants. MAPK3 and MAPK6 play a critical role in promoting cell division in integuments specifically during ovule development as well as being involved in abiotic stress response [5]. MAPK4, MAPK5, MAPK6 and MAPK7 were specifically induced by abiotic stress treatments such as salt, drought and low temperature in maize [6]. *GhMAPK16* might be relevant to abiotic stress signal transduction pathways and its over-expression in *Arabidopsis* led to significant tolerance to drought stress [7]. The MAPK cascade gene family has been well studied in rice, maize and *Brachypodium distachyon* [8,9,10]. In wheat, the genome organization, evolutionary features and expression profiles of the MAP3K gene family has also been systematically studied [4]. However, information on wheat MAPK and MAPKK gene families is not well-understood, especially those families involved in abiotic stress response.

In this study, 54 MAPK and 18 MAPKK genes were identified in wheat genome based on a bioinformatics search. The phylogenetic tree, conserved motifs, gene expression pattern and regulatory network of these MAPKs and MAPKKs were further systematically analyzed. Phylogenetic tree and conserved motifs analysis of wheat MAPK and MAPKK families classified them into four groups. Additionally, the expression patterns of 54 MAPK and 18 MAPKK genes under abiotic stress or in various tissues were comprehensively investigated by RNA-seq analyses. Finally, the interaction network of putative wheat MAPK and MAPKK genes was also investigated. Overall, this study provided basic information about the genomic organization of MAPK and MAPKK genes in wheat, which will facilitate further functional studies.

## 2. Materials and Methods

### 2.1. Identification of MAPK and MAPKK Genes in Wheat

Potential members of wheat MAPK and MAPKK gene families were identified according to the method described by Wang et al [4]. All available protein sequences for wheat genotype Chinese Spring (CS42) were downloaded from the Ensemble database [11]. The available MAPK and MAPKK genes in *Arabidopsis thaliana*, *Oryza sativa* and *B. distachyon* were used to construct an hidden Markov model (HMM) profile using the hmm-build tool embedded in HMMER 3.0, and then the HMM profile was used to search wheat proteins using the hmmsearch tool embedded in HMMER 3.0 [12]. Conserved domains of wheat MAPK and MAPKK members were confirmed by PFAM [13] and InterProScan database [14]. Finally, all sequences obtained were verified by a BLASTN (Nucleotide BLAST) similarity search against wheat expressed sequence tags (ESTs) deposited in the National Center for Biotechnology Information NCBI database. The theoretical pI (isoelectric point) and Mw (molecular weight) of candidate genes were calculated using compute pI/Mw tool online (http://web.expasy.org/compute_pi/). Subcellular localization of these genes was predicted using the CELLO v2.5 web server [15].

### 2.2. Multiple Alignments and Phylogenetic Analysis

Multiple sequence alignments were performed using ClustalW [16]. An unrooted phylogenetic tree was generated by MEGA 6.0 software using the neighbor-joining method and the bootstrap test method with 1000 replications [17]. The MEME program [18] was used to predict the conserved motifs of *TaMAPKs* and *TaMAPKKs*.

### 2.3. Analysis of the Expression Profiles of MAPK and MAPKK Genes by RNA-seq Datasets

To study the expression of MAPK and MAPKK genes in different organs and response to stresses, a total of 28 RNA-seq datasets of wheat variety Chinese Spring obtained from WHEAT URGI (https://urgi.versailles.inra.fr/files/RNASeqWheat/) and NCBI Sequence Read Archive (SRA) database were used to investigate differential expression of *TaMAPK* and *TaMAPKK* genes (Table S1). TopHat and Cufflinks software were used to analyze gene expression based on these RNA-seq data with the default parameters [19]. The FPKM (fragments per kilobase of transcript per million fragments mapped) value was calculated for each gene, and the log10-transformed (FPKM + 1) values of MAPK and MAPKK genes were used to generate heat maps.

### 2.4. Co-expression Network Analysis

Correlation networks are increasingly being used in bioinformatics applications. The WGCNAR_1.49 package was used to construct the co-expression network of wheat MAPK and MAPKK genes by performing weighted correlation network analysis of RNA-seq data [20].

## 3. Results and Discussion

### 3.1. Genome-wide Identification of the MAPK and MAPKK Gene Families in Wheat

MAPK and MAPKK gene families are the important protein kinases involved in many physiological processes [21]. In order to identify members of the two families, we performed a HMM search and a total of 54 non-redundant MPAK and 18 MAPKK genes were identified in the wheat genome (Table 1). The predicted genes in the two families were named *TaMAPK1* to *TaMAPK54* and *TaMAPKK1* to *TaMAPKK18*, respectively. The number of MAPK genes in wheat (54) was much higher than in rice (16), maize (19) and *B. distachyon* (16) [8,9,10]. The number of *TaMAPKK* (18) was also slightly higher than in *B. distachyon* (12) and cucumber (14) [22]. The locations of both groups of MAPK cascade genes were not random across the wheat chromosomes. Sixteen of the *TaMAPK* genes were located on homoeologous group 7 chromosomes, 14 were located in group 1 chromosomes, and 9, 7, 6 and 2 were in groups 6, 3, 4 and 5, respectively. There was no MAPK gene on a group 2 chromosome. The locations of *TaMAPKK* genes differed from *TaMAPK*s. Homologous group 4 contained the most *TaMAPKK* genes, at 10, followed by group 5 with 5 *TaMAPKK*s, then groups 3 and 6, harboring 2 and 1, respectively. No *TaMAPKK* gene was present in groups 1, 2 and 7.

To support the actual existence of these putative genes, we performed a BLASTN search against the wheat EST and UniGene database using the MAPK and MAPKK genes as queries (Table 1). Fifty-one MAPK genes and 5 MAPKK genes had EST support, representing 94.4% and 27.8% of the predicted genes, respectively. We speculated that the *TaMAPKs* and *TaMAPKK*s with no EST support might have very low expression that could not be detected experimentally, or was not expressed under any conditions we used. The lengths of putative *TaMAPK* proteins ranged from 184 to 598 amino acids, with putative molecular weights (Mw) ranging from 21.4 to 68.2 kDa and theoretical pI ranging from 5.19 to 9.17, respectively. The lengths of *TaMAPKK* proteins ranged from 188 to 647 amino acids, Mw from 20.7 to 71.6 kDa and pI from 5.37 to 8.95, respectively. Subcellular localization analysis found that most of *TaMAPKs* and *TaMAKKs* were localized in cytoplasmic (Table 1).

### 3.2. Multiple Alignments, Phylogenetic and Conserved Motif Analysis of TaMAPKs and TaMAPKKs

**Figure 1:**
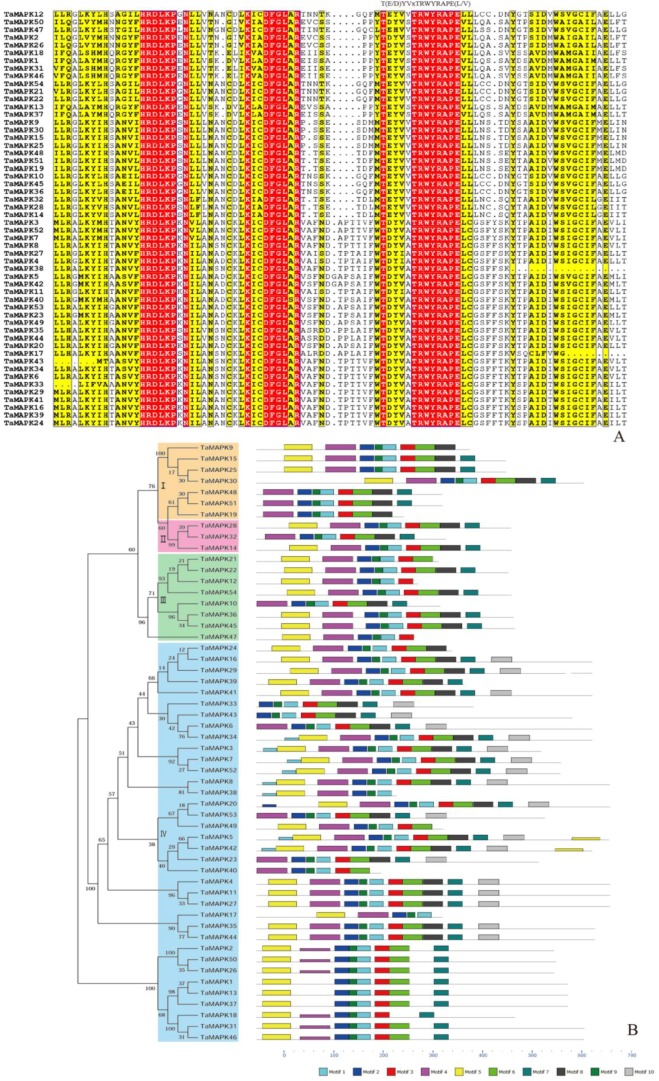
Phylogenetic relationship and conserved motifs composition of the wheat MAPK family. (**A**) Multiple alignments to identify the conserved signature motif T(E/D)YVxTRWYRAPE(L/V) in the protein sequences of wheat MAPK family; (**B**) Phylogenetic analysis (left) and conserved domain (right) compositions of wheat MAPK family. The unrooted phylogenetic tree was constructed using MEGA 6.0 tool with the neighbor-joining method and 1,000 bootstrap replications. Conserved motifs were predicated by MEME tool and each motif was represented with different colored boxes.

To further evaluate the phylogenetic relationships of the wheat MAPK and MAPKK genes, the full-length protein sequences of the 54 *TaMAPK*s and 18 *TaMAPKK*s were aligned using ClustalW software [16] and phylogenetic trees were constructed using the neighbor joining (NJ) method in MEGA 6.0 [17]. As shown in Figure 1A, the sequences of the *TaMAPK* genes in the motif regions were highly conserved. Through phylogenetic analyses relative to homologs in *Arabidopsis* and cucumber, we classified the *TaMAPK*s into four groups (Figure 1B), each group including 7 (Group I), 3 (II), 8 (III), 36 (IV) members. The results indicated evolutionary differences in wheat MAPK genes, consistent with those in *B. distachyon* [8]. Each MAPK cluster classified by phylogenetic analysis shared similar conserved motif compositions. A total of ten motifs were identified in *TaMAPK* proteins. Motif TXY is an important phosphorylation site for MAPK activation. Groups I-III have a TEY motif in their activation loops, except *TaMAPK47* where THE replaced the TEY. The THE motif was not found in rice, maize, *M. domestica* or *B. distachyon* [9,10,23,24] suggesting that it might be wheat-specific.

**Figure 2:**
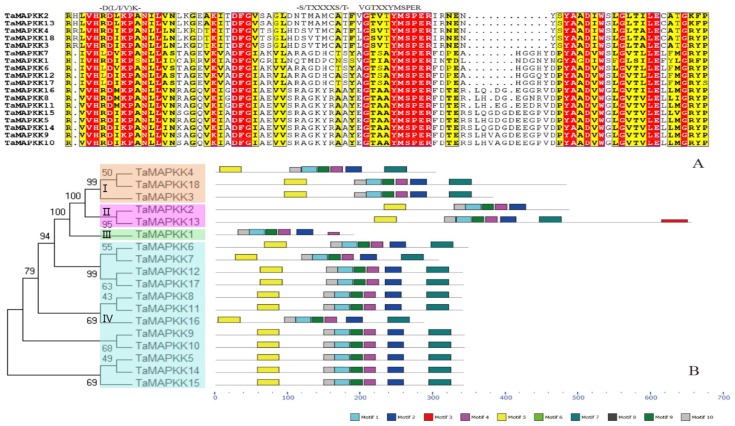
Phylogenetic relationship and conserved motifs compositions of the wheat MAPKK family. (**A**) Multiple alignments and conserved signature motifs found in the protein sequences of wheat MAPKK family; (**B**) Phylogenetic analysis (left) and conserved domain compositions (right) in wheat MAPKK family. The unrooted phylogenetic tree was constructed by MEGA 6.0 using the neighbor-joining method and the bootstrap test method with 1000 replications. Conserved motifs were predicated by MEME tool and each motif was represented with different colored boxes.

As shown in Figure 2A, members of the *TaMAPKK* family also clustered into four groups. Groups I, II and III contained a S/T-X5-S/T motif, which functions as a phosphorylation site, but was not present in Group IV. This motif is not only found in rice, *Arabidopsis* and *B. distachyon*, but also in some animals and fungi [24]. Most interestingly, the MAPKKs that include the S/T-X5-S/T motif were highly expressed in many tissues or responded to multiple stresses.

### 3.3. Tissue-Specific Expression Patterns of TaMAPK and TaMAPKK Genes

**Figure 3:**
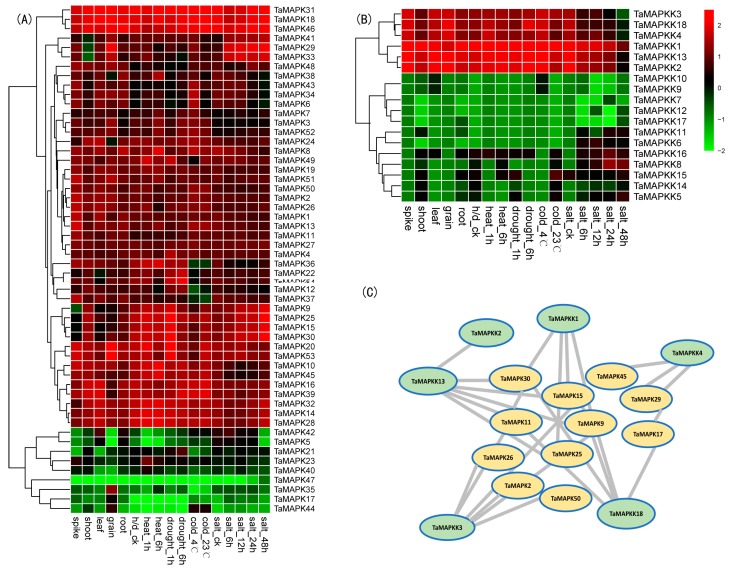
Expression patterns and interaction network of MAPK/MAPKK genes in wheat. (**A**) Relative expression levels of MAPK genes in various tissues and under different abiotic stresses determined using RNA-seq. Red represents increased and green represents decreased expression level; (**B**) Relative expression level of wheat MAPKK genes in various tissues and under abiotic stresses determined using RNA-seq; (**C**) The interaction network of MAPK/MAPKK genes in wheat.

The expression patterns of *TaMAPKs* in various wheat tissues were investigated using RNA-seq data for five tissues. As shown in Figure 3A, the heat map indicates that all 54 detected genes were involved in various biological processes and were expressed in the majority of wheat tissues, but their expression levels were highly variable. *TaMAPK14*, *18*, *28*, *31*, *32* and *46* were expressed at high levels in all five tissues. Some *TaMAPKs* were highly expressed in specific tissues. For example, *TaMAPK17* and *35* were highly expressed in developing grain, suggesting that those genes may play indispensable roles in grain development. *TaMAPK5* and *42* were relatively highly expressed in leaf and root tissues. *TaMAPK*s highly expressed in specific organs may have specific roles in growth and development of the corresponding tissues. It is noteworthy that tissue expression specificity of MAPK genes has been reported in *B. distachyon*, maize and cucumber [9,15,24]. All members of group II were highly expressed in multiple tissues, consistent with *CsMAPK13* in cucumber and *BdMAPK11* in *B. distachyon* [15,24].

Expression profiles of *TaMAPKK* genes in different tissues showed that most genes were expressed in five different tissues. Interestingly, MAPKKs with the S/T-X5-S/T motif show high expression in specific tissues (Figure 3B). For example, *TaMAPKK9* and *TaMAPKK10* showed relatively higher expression in leaves than in other tissues, indicating that they positively controlled leaf development. Members of Groups I, II and III with S/T-X5-S/T motifs had higher expression in all organs, consistent with *BdMAPKK3* and *BdMAPKK6* in *B. distachyon* and suggesting that this motif was an important phosphorylation site in relation to gene expression level [24]. Conserved domains identification and analysis may facilitate identification of functional units in these kinase genes and accelerate an understanding of their roles in plant growth and development as well as stress response.

### 3.4. Expression Patterns of TaMAPK and TaMAPKK Genes under Abiotic Stress

There is increasing data showing that the MAPK and MAPKK gene families have key roles in determining response to abiotic stresses [21]. For example, *ZmMAPK1*, *GhMAPK4* and *SiMAPK7* were reported to regulate abiotic stress response [25,26,27]. We analyzed the expression patterns of *TaMAPK* genes to determine whether they were responsive to heat, drought, cold or salt stress. As shown in Figure 3A, 84% and 16% *TaMAPKs* displayed up- or down-regulation following stress treatments. *TaMAPK38* was up-regulated after 6 h of heat stress, whereas *TaMAPK5* and *TaMAPK42* were suppressed. *TaMAPK10* and *TaMAPK45* were suppressed whereas *TaMAPK29*, *TaMAPK33* and *TaMAPK41* were induced by salt stress. This was consistent with previous research reporting that *TaMAPK4* was strongly induced under high salinity stress [28], and that over-expression of *OsMAPK33* in rice led to enhanced salt sensitivity [29]. However, multiple numbers of clustered genes, such as *TaMAPK32*, *TaMAPK14* and *TaMAPK28*, had similar expression profiles under abiotic stress, indicating that these genes might have analogous physiological functions. The expression levels of clustered genes were not significantly altered by stress treatment as also found in *B. distachyon* [24]. Finally, the *TaMAPK47* gene was specifically expressed after 24 h of salt treatment, indicating it might be a salt-responsive candidate. Genes that are expressed under abiotic stress conditions may be the specific genes responsible for regulation of stress response and tolerance. Compared with *TaMAPK* genes *TaMAPKK*s show more significant differences under various stress conditions. For example, transcripts of *TaMAPKK8* and *TaMAPKK15* were down-regulated under heat treatment for 1 h, but were induced after 6 h. This was contrary to reports for *SbMAPKK* in *S. brachiata* in which there was up-regulation followed by down-regulation under dehydration treatment [30]. *TaMAPKK12* and *16* were induced and *TaMAPKK7* and *17* were reduced under all stress treatments, whereas other genes were reduced or induced following just one or two stress treatments. *TaMAPKK5* and *TaMAPKK14* showed opposite expression patterns following heat and salt treatments, suggesting that functional differentiation had occurred in these genes and that they were involved in regulating different stress signaling pathways. In summary, wheat MAPK/MAPKK genes likely play vital roles in different biological process and various abiotic stresses.

### 3.5. Interactions between TaMAPK and TaMAPKK Family Members

In general, MAPK cascade kinase genes form conserved signaling modules, involving three functionally linked protein kinases: MAPKs, MAPKKs and MAP3Ks [8]. In this study, we constructed the regulatory network of the MAPK, MAPKK cascade based upon different tissues and stress treatments in wheat. The WGCNAR package [20] provides a comprehensive set of functions for performing weighted correlation network analysis by RNA-seq data. As shown in Figure 3C, 6 MAPKK genes and 11 MAPK genes were involved in a single cascade. The 6 MAPKK genes contained S/T-X5-S/T motifs showed high expression levels. Furthermore, 4 of the 11 MAPK genes in Group I, including *TaMAPK9*, *TaMAPK15*, *TaMAPK25* and *TaMAPK30*, showed close associations with MAPKK genes. In *Arabidopsis*, Group I members *AtMAPK3* and *AtMAPK6* play important roles in anther cell differentiation and normal anther lobe formation, and *AtMAPK6* is involved in seed formation and the modulation of lateral and primary root development [5,31], suggesting that *TaMAPK9*, *TaMAPK15*, *TaMAPK25* and *TaMAPK30* might be involved in the regulatory network that controls organ differentiation or development. The constructed interaction network between *TaMAPK* and *TaMAPKK* provides information for further studies on MAPK regulatory pathways in wheat and other species.

## Figures and Tables

**Table 1:** Characteristics of putative wheat mitogen-activated protein kinase (MAPK) and MAPK kinase (MAPKK) genes.

Gene Family	Gene	pI	Mw (kDa)	Amino Acid Length	EST Count	Ensemble Wheat Gene ID	Subcellular Location
MAPK	*TaMAPK1*	9.71	51.6	460	25	Traes_6BL_BF59BFB93	Periplasmic
	*TaMAPK2*	5.89	50.3	440	10	Traes_4AL_6F3D0ACCA	Cytoplasmic
	*TaMAPK3*	8.83	48.4	421	2	Traes_7AL_F5620757F	Cytoplasmic
	*TaMAPK4*	8.85	62.8	553	24	Traes_1DS_66BD773BF	Cytoplasmic
	*TaMAPK5*	8.51	59.1	521	1	Traes_1DL_52D511CDD	Cytoplasmic
	*TaMAPK6*	9.48	57.5	519	12	Traes_1BL_7FA80EF00	Periplasmic
	*TaMAPK7*	8.34	51.9	450	1	Traes_7DL_FE0ECD387	Cytoplasmic
	*TaMAPK8*	8.94	68.2	598	33	Traes_3DL_5D82311EA	Cytoplasmic
	*TaMAPK9*	6.41	36.3	315	35	Traes_4BS_9285C0809	Cytoplasmic
	*TaMAPK10*	5.7	31.2	272	22	Traes_6BS_17C1E5829	Cytoplasmic
	*TaMAPK11*	8.74	62.3	549	26	Traes_1AS_C9CFD7AC8	Cytoplasmic
	*TaMAPK12*	8.78	26.7	239	19	Traes_7AL_4AF13CC8B	Cytoplasmic
	*TaMAPK13*	9.71	51.6	460	28	Traes_6DL_F48A5E31E	Periplasmic
	*TaMAPK14*	5.75	42.9	377	36	Traes_1AL_89DDB4243	Cytoplasmic
	*TaMAPK15*	5.46	42.8	369	61	Traes_4BL_2CEFDE904	Cytoplasmic
	*TaMAPK16*	9.32	67.2	586	22	TRAES3BF058500020CFD_g	Periplasmic Cytoplasmic
	*TaMAPK17*	8.2	31.4	275	0	Traes_6BS_2D0054D1F	InnerMembrane Cytoplasmic
	*TaMAPK18*	9.51	44.3	382	9	Traes_7DS_C268073F4	Periplasmic Cytoplasmic
	*TaMAPK19*	6.13	25.5	218	5	Traes_7DS_1D8A8BFA2	Cytoplasmic
	*TaMAPK20*	7.05	65.1	578	57	Traes_7DL_FB75EA9C3	Cytoplasmic
	*TaMAPK21*	8.82	30.8	269	22	Traes_7DL_99CBA4440	Cytoplasmic
	*TaMAPK22*	7.19	42.7	373	25	Traes_7DL_73DF29BF0	Cytoplasmic
	*TaMAPK23*	7.24	47.9	417	1	Traes_3DL_281B6BCBF	Cytoplasmic
	*TaMAPK24*	9.18	33.4	289	9	Traes_3DL_38B762939	Cytoplasmic
	*TaMAPK25*	5.46	42.8	369	61	Traes_4DL_15045954F	Cytoplasmic
	*TaMAPK26*	5.55	50.1	440	10	Traes_5DL_8DC610F26	Cytoplasmic
	*TaMAPK27*	8.92	62.4	549	25	Traes_1BS_26C55B2B1	Cytoplasmic
	*TaMAPK28*	5.75	42.9	376	36	Traes_1BL_C5CD09285	Cytoplasmic
	*TaMAPK29*	8.99	52.5	457	2	Traes_1BL_9360D8CEC	Cytoplasmic
	*TaMAPK30*	6.5	55.4	484	63	Traes_4AS_5015DF7A2	Cytoplasmic
	*TaMAPK31*	9.5	54.9	485	66	Traes_4AL_E432524A0	Periplasmic
	*TaMAPK32*	5.19	32.8	280	28	Traes_1DL_319058D6B	Cytoplasmic
	*TaMAPK33*	9.25	36.3	321	1	Traes_1DL_8C6B737E9	Periplasmic
	*TaMAPK34*	9.01	61.0	539	27	Traes_1DL_73AAC8631	Cytoplasmic
	*TaMAPK35*	6.36	57.2	500	0	Traes_6AS_50BE5D59F	Cytoplasmic
	*TaMAPK36*	7.2	43.5	380	22	Traes_6AS_8225741A6	Cytoplasmic
	*TaMAPK37*	9.71	51.5	460	28	Traes_6AL_BE97161B9	Periplasmic
	*TaMAPK38*	8.27	24.1	207	17	Traes_3AL_F88B0A8E6	Cytoplasmic
	*TaMAPK39*	8.99	35.5	307	21	Traes_3AL_669FE0293	Cytoplasmic
	*TaMAPK40*	6.2	21.4	184	1	Traes_3AL_532CA9EE7	Cytoplasmic
	*TaMAPK41*	9.14	61.7	543	3	Traes_1AL_30C4B017F	Cytoplasmic
	*TaMAPK42*	8.52	56.4	496	2	Traes_1AL_14DCD6020	Cytoplasmic
	*TaMAPK43*	9.5	51.6	467	7	Traes_1AL_A7173BBAE	Periplasmic
	*TaMAPK44*	6.48	57.5	500	1	Traes_6DS_6424C38F2	Cytoplasmic
	*TaMAPK45*	7.63	43.8	382	21	Traes_6DS_A61864DB2	Cytoplasmic
	*TaMAPK46*	9.46	55.1	484	60	Traes_7AS_E1135D559	Periplasmic Cytoplasmic
	*TaMAPK47*	8.74	26.9	232	0	Traes_7AS_71B4C13A6	Cytoplasmic
	*TaMAPK48*	5.31	31.9	275	8	Traes_7AS_74B19D5B6	Cytoplasmic
	*TaMAPK49*	6.55	31.6	276	15	Traes_7AL_81A545A54	Cytoplasmic
	*TaMAPK50*	6.04	50.4	443	10	Traes_5BL_A39EC2FCD	Cytoplasmic
	*TaMAPK51*	5.31	31.9	275	8	Traes_7BS_F5BB0A116	Cytoplasmic
	*TaMAPK52*	8.5	51.5	449	2	Traes_7BL_44A22C7FC	Cytoplasmic
	*TaMAPK53*	6.55	48.3	426	52	Traes_7BL_992E1F443	Cytoplasmic
	*TaMAPK54*	7.2	43.2	377	29	Traes_7BL_95B1705A2	Cytoplasmic
MAPKK	*TaMAPKK1*	5.91	20.7	188	4	Traes_6DL_8D2D914D7	Periplasmic Cytoplasmic
	*TaMAPKK2*	7.88	53.5	480	0	Traes_5BL_BB574E77D	Cytoplasmic
	*TaMAPKK3*	5.37	41.9	377	3	Traes_5DL_0886CB561	Cytoplasmic
	*TaMAPKK4*	5.89	33.6	299	1	Traes_5AL_5EFCEDAFB	Cytoplasmic
	*TaMAPKK5*	7.7	36.2	337	0	Traes_4BS_EE048CC01	Cytoplasmic
	*TaMAPKK6*	8.95	36.2	343	0	Traes_4BS_59F3F68D1	Periplasmic Cytoplasmic
	*TaMAPKK7*	8.28	31.8	303	0	Traes_4BS_84D913A2E	Outer Membrane Periplasmic Cytoplasmic
	*TaMAPKK8*	8.62	35.9	334	0	Traes_4BS_BDB56BC90	Cytoplasmic
	*TaMAPKK9*	7.72	36.3	338	0	TRAES3BF024700110CFD_g	Cytoplasmic
	*TaMAPKK10*	7.72	36.3	338	0	TRAES3BF024900030CFD_g	Cytoplasmic
	*TaMAPKK11*	6.9	36.2	336	0	Traes_4DS_7F2F2671B	Cytoplasmic
	*TaMAPKK12*	8.82	35.4	336	0	Traes_4DS_7A016C2E4	Periplasmic Cytoplasmic
	*TaMAPKK13*	6.69	71.6	647	1	Traes_5DL_F89F21E65	Cytoplasmic
	*TaMAPKK14*	7.15	36.1	337	0	Traes_4AL_75E7BE9EE	Cytoplasmic
	*TaMAPKK15*	8.21	36.3	337	0	Traes_4AL_429683D72	Cytoplasmic
	*TaMAPKK16*	8.36	30.3	283	0	Traes_4AL_B7C432896	Cytoplasmic
	*TaMAPKK17*	8.88	35.6	337	0	Traes_4AL_84DFF6A541	Cytoplasmic
	*TaMAPKK18*	5.64	53.8	476	3	Traes_5BL_EC9896AB3	Cytoplasmic

EST, expressed sequence tag; pI, isoelectric point; Mw, molecular weight

## Supplementary Materials

The following can be found online at www.mdpi.com/2073-4425/8/10/284/s1, Table S1. Accession number and sample information of RNA-seq datasets used in this study.
